# Supplementary material for: The NGF R100W Mutation, Associated with Hereditary Sensory Autonomic Neuropathy Type V, Specifically Affects the Binding Energetic Landscapes of NGF and of Its Precursor proNGF and p75NTR
Source: Biology (Basel). 2023 Feb 25;12(3):364. doi: 10.3390/biology12030364 (PMC10045213; doi:10.3390/biology12030364)
Supplement: Supplementary file 1 [file biology-12-00364-s001.zip › biology-2189732-supplementary.pdf]

Supplementary Materials and Methods

Surface Plasmon Resonance (SPR)

SPR measurements were performed using a BIACore 2000 instrument (BIACore AB, Uppsala, Sweden). The extracellular domains of TrkA and p75NTR, expressed in baculovirus-infected insect cells as camel immunoadhesins [40 in main text], were immobilized on a CM5 sensor chip by cross-linking the amine groups according to the manufacturer's instructions, obtaining SPR signals, after completion of the chip regeneration cycle, of 8860 resonance units (RU) and of 4320 RU, respectively. The binding kinetics were determined by injection in PBS buffer with addition of 0.005% v/v Surfactant P20 of hNGF R100W (in the 4–500 nM concentration range) at a flow rate of 30 ul/min. Data were analysed using the BIA evaluation 3.0 package (GE Healthcare) to yield the apparent equilibrium constant K_D (defined as the ka/kd ratio). The experiment was performed in triplicate.

Table S1: Details of NGF residues involved in the interface surface of the NGF/p75NTR complex (PDB_ID 1SG1)

NGF Chain: residue	Accessible Surface Area ASA(Å^2)	Buried Surface Area BSA(Å^2) ; Buried area percentage, one bar per 10%	Solvation energy effect Δ^iG (kcal/mol)	RMSF NGF WT (Å)	RMSF NGF R100W (Å)
A:ASP 30	4.04	2.08 ||||||	-0.04	0.971	1.081
A:ILE 31	87.78	46.67 ||||||	0.75	0.958	1.067
**A:LYS 32***	**176.25**	**78.12 |||||**	**-0.82**	**1.076**	**1.354**
A:LYS 34	88.67	2.34 |	0.04	1.219	1.542
A:ASP 72	68.34	1.23 |	-0.02	1.284	1.347
A:HIS 75	88.29	37.20 |||||	0.22	1.191	1.234
A:TRP 76	97.47	55.47 ||||||	0.81	1.089	1.11
A:PHE 86	79.04	1.25 |	0.02	0.925	0.88
**A:LYS 88***	**110.11**	**33.20 ||||**	**-0.54**	**0.887**	**0.97**
A:ASP 93	44.17	9.84 |||	-0.13	1.713	2.015
A:LYS 95	177.77	3.18 |	0.05	2.293	2.795
**A:GLN 96***	**118.92**	**78.68 |||||||**	**-0.23**	**1.628**	**2.168**
A:ALA 97	47.83	3.07 |	-0.04	1.236	1.652
A:ALA 98	34.30	26.10 ||||||||	0.42	1.09	1.358
A:TRP 99	136.23	43.96 ||||	0.05	1.05	1.134
A:ARG 100	65.43	58.0 ||||||||||	-1.33	0.939	1.003
A:PHE 101	88.28	44.05 |||||	0.69	0.888	0.927
A:ARG 114	191.36	113.17 ||||||	-1.52	1.735	1.471
B:ARG 9	246.58	24.61 |	0.04	3.955	2.216
B:GLY 10	67.81	30.55 |||||	0.26	2.278	1.631
B:GLU 11	116.23	1.23 |	-0.01	1.268	1.321
B:PHE 12	173.89	86.14 |||||	1.38	0.812	0.858
B:ASP 16	42.50	10.07 |||	0.00	0.818	0.916
B:SER 17	54.87	3.31 |	-0.04	0.955	0.945
B:VAL 18	75.58	32.13 |||||	0.51	0.871	0.981
B:VAL 20	82.60	6.58 |	0.11	0.942	1.051
**B:TRP 21***	**162.16**	**81.48 ||||||**	**0.80**	**0.998**	**0.982**
B:SER 47	46.10	9.84 |||	0.15	3.009	2.6
B:VAL 48	102.73	40.44 ||||	0.25	2.289	2.123

B:PHE 49	112.90	54.09 |||||	0.87	1.641	1.546
**B:LYS 50***	**96.17**	**42.05 |||||**	**0.40**	**1.115**	**1.202**
**B:TYR 52***	**120.88**	**70.96 ||||||**	**0.54**	**1.003**	**0.876**
**B:ARG 59***	**154.55**	**44.36 |||**	**-0.44**	**0.854**	**1.003**
**B:ARG 69***	**120.95**	**75.10 ||||||||**	**-0.68**	**0.847**	**0.937**
B:GLY 70	82.13	0.15 |	-0.00	0.992	1.052

*Residues involved in hydrogen bonds or salt bridges

Table S2: Details of p75NTR residues involved in the interface surface of the NGF/p75NTR complex (PDB_ID 1SG1)

P75NTR Chain: residue	Accessible Surface Area ASA(Å^2)	Buried Surface Area BSA(Å^2) ; Buried area percentage, one bar per 10%	Solvation energy effect $\Delta^i G$ (kcal/mol)	RMSF p75NTR with NGF WT (Å)	RMSF p75 NTR with NGF R100W (Å)
X:PRO 38	87.55	2.52 |	0.04	1.086	1.354
X:LEU 40	113.34	22.93 |||	0.37	0.94	1.303
**X:ASP 41***	**87.70**	**67.28 ||||||||**	**-0.05**	**0.883**	**1.191**
**X:ASN 42 ***	**104.49**	**49.22 |||||**	**-0.20**	**0.924**	**1.193**
X:MET 67	108.01	57.99 ||||||	1.22	1.124	1.28
**X:SER 68***	**44.34**	**18.08 |||||**	**-0.07**	**1.091**	**1.208**
X:ALA 69	28.52	0.17 |	0.00	0.989	1.108
X:PRO 70	87.17	48.74 ||||||	0.78	0.924	1.126
X:CYS 71	7.12	1.75 |||	-0.02	0.82	1.189
X:GLU 73	70.96	36.80 ||||||	-0.30	0.788	1.214
**X:TYR 83***	**169.58**	**59.05 ||||**	**0.55**	**1.175**	**1.462**
X:LEU 106	29.52	21.13 ||||||||	0.14	1.366	1.35
X:VAL 107	60.33	31.56 ||||||	-0.07	1.682	1.69
X:PHE 108	127.71	45.71 ||||	0.73	1.751	1.669
X:GLU 119	71.50	0.25 |	-0.00	1.608	1.685
**X:VAL 133***	**78.66**	**50.11 |||||||**	**0.48**	**1.318**	**1.405**
**X:ASP 134***	**54.66**	**14.49 |||**	**-0.25**	**0.861**	**1.273**
X:PRO 135	63.99	49.08 ||||||||	0.79	0.908	1.316
X:LEU 137	81.35	16.42 |||	0.26	1.194	1.659
X:ASN 20	72.79	18.47 |||	0.15	1.147	1.752
**X:LEU 21***	**104.99**	**74.71 ||||||||**	**0.97**	**1.066**	**1.486**
**X:ASP 41***	**87.70**	**20.42 |||**	**-0.20**	**0.883**	**1.191**
X:VAL 49	98.21	16.24 ||	0.26	1.265	1.815
X:SER 68	44.34	12.03 |||	-0.14	1.124	1.28
X:ALA 69	28.52	24.36 |||||||||	0.39	1.091	1.208
X:PRO 70	87.17	38.43 |||||	0.59	0.989	1.108
X:CYS 71	7.12	0.61 |	-0.01	0.924	1.126
X:VAL 72	72.30	67.78 ||||||||||	1.08	0.82	1.189
X:GLU 73	70.96	19.53 |||	-0.25	0.804	1.236
X:ALA 74	33.20	30.50 ||||||||||	0.38	0.788	1.214
**X:ASP 75***	**85.99**	**61.47 ||||||||**	**-0.50**	**0.85**	**1.275**
**X:ASP 76***	**43.30**	**7.93 ||**	**-0.00**	**0.828**	**1.213**
X:VAL 78	79.94	29.04 ||||	0.46	0.993	1.192
X:LEU 106	29.52	0.86 |	-0.01	1.366	1.35

**X:GLU 119***	**71.50**	**17.35 |||**	**-0.20**	**1.608**	**1.685**
X:PRO 122	70.58	13.32 ||	0.21	1.852	1.831
X:THR 125	26.72	14.32 ||||||	0.23	1.463	1.738
X:PRO 135	63.99	14.91 |||	0.24	0.908	1.316
**X:CYS 136***	**19.96**	**19.96 |||||||||||**	**-0.12**	**1.018**	**1.297**
X:LEU 137	81.35	11.22 ||	0.18	1.194	1.659
X:PRO 138	107.24	74.89 |||||||	1.20	1.533	2.008
X:CYS 139	34.40	5.41 ||	-0.06	1.727	2.199
X:VAL 141	133.59	0.49 |	0.01	2.506	3.188

*Residues involved in hydrogen bonds or salt bridges

Table S3: Details of proNGF residues involved in the interface surface of the proNGF/2p75NTR complex (PDB_ID 3IJ2)

proNGF Chain: residue	Accessible Surface Area ASA(Å^2)	Buried Surface Area BSA(Å^2) ; Buried area percentage, one bar per 10%	Solvation energy effect Δ^iG (kcal/mol)	RMSF proNGF WT (Å)	RMSF proNGF R100W (Å)
A:HIS 8	143.87	16.35 ||	-0.11	2.18	7.354
A:MET 9	171.03	33.02 ||	0.01	1.621	4.724
A:GLY 10	62.06	26.64 |||||	0.22	1.202	2.224
A:GLU 11	98.86	2.70 |	-0.03	0.926	1.276
A:PHE 12	159.19	73.12 |||||	1.17	1.034	1.073
A:ASP 16	29.78	11.89 ||||	0.03	0.776	0.909
A:SER 17	57.57	0.74 |	-0.01	0.865	0.855
A:VAL 18	91.42	40.88 |||||	0.65	0.861	0.869
**A:TRP 21***	**157.06**	**83.93 ||||||**	**0.85**	**0.968**	**0.99**
A:ASN 45	125.57	5.62 |	0.01	4.097	4.705
A:ASN 46	156.75	0.47 |	0.01	3.641	4.138
A:SER 47	41.20	38.68 ||||||||||	0.62	2.429	2.851
A:VAL 48	80.35	27.87 ||||	0.40	1.842	2.045
A:PHE 49	116.17	48.03 |||||	0.40	1.475	1.734
A:ARG 50	119.23	5.28 |	-0.06	1.117	1.14
**A:TYR 52***	**131.06**	**75.25 ||||||**	**0.75**	**0.941**	**0.94**
**A:ARG 59***	**145.26**	**46.46 ||||**	**0.12**	**0.841**	**0.878**
A:PRO 63	17.34	5.89 ||||	-0.07	1.005	1.292
A:VAL 64	52.02	21.77 |||||	0.35	1.114	1.468
A:GLU 65	162.07	16.50 ||	-0.24	1.433	1.466
**A:ARG 69***	**86.95**	**63.47 ||||||||**	**-0.56**	**0.77**	**1.06**
A:LYS 74	149.74	6.87 |	0.11	1.157	1.555
A:ILE 31	77.91	43.75 ||||||	0.70	0.976	0.998
**A:LYS 32***	**172.24**	**75.05 |||||**	**-0.66**	**1.135**	**1.159**
A:LYS 34	86.05	0.13 |	-0.00	1.307	1.52
A:ASP 72	69.74	0.24 |	-0.00	0.89	1.194
A:LYS 74	149.74	14.61 |	-0.54	1.157	1.555
A:HIS 75	92.83	40.37 |||||	0.16	1.043	1.413
A:TRP 76	80.01	49.60 |||||||	0.65	0.879	1.184
**A:LYS 88***	**126.65**	**37.89 |||**	**-0.28**	**0.849**	**0.852**
A:GLN 96	117.54	62.13 ||||||	0.43	2.103	2.127

A:ALA 98	55.50	30.02 ||||||	0.48	1.352	1.44
A:TRP 99	152.05	38.31 |||	0.06	1.128	1.189
A:ARG 100	73.13	52.20 |||||||||	-0.93	0.993	1.039
A:PHE 101	82.24	43.00 ||||||	0.69	0.862	0.887
**A:ARG 114***	**187.23**	**118.19 ||||||||**	**-2.21**	**1.39**	**1.399**
A:THR 117	189.41	7.07 |	0.10	5.041	3.516
B:HIS 8	223.06	15.19 |	0.11	3.055	2.978
B:MET 9	166.57	22.48 ||	-0.07	2.054	2.07
B:GLY 10	53.15	29.02 ||||||	0.08	1.48	1.601
B:GLU 11	134.45	0.96 |	-0.01	0.955	1.338
B:PHE 12	164.58	78.75 |||||	1.26	0.803	1.031
B:ASP 16	29.79	11.38 ||||	0.00	0.819	0.798
B:SER 17	58.11	3.07 |	-0.04	0.808	0.884
B:VAL 18	91.54	45.83 ||||||	0.73	0.88	0.914
B:VAL 20	77.86	0.67 |	0.01	0.961	1.008
**B:TRP 21***	**157.20**	**93.58 ||||||**	**0.92**	**0.941**	**0.939**
B:ASN 45	124.42	0.24 |	-0.00	4.223	5.089
B:SER 47	42.56	36.82 ||||||||||	0.59	3.41	3.568
B:VAL 48	79.81	25.52 ||||	0.36	2.43	2.203
B:PHE 49	117.31	45.50 ||||	0.35	2.026	1.45
B:ARG 50	120.68	3.31 |	-0.04	1.38	1.002
B:TYR 52	128.93	70.58 ||||||	0.76	0.96	0.879
B:PHE 54	95.90	0.31 |	0.00	0.813	0.828
**B:ARG 59***	**143.76**	**45.33 ||||**	**0.08**	**0.899**	**0.871**
B:PRO 63	16.84	7.23 |||||	-0.08	0.879	1.019
B:VAL 64	52.95	22.90 |||||	0.37	0.948	1.2
B:GLU 65	162.46	24.27 ||	-0.33	1.205	1.59
**B:ARG 69***	**87.15**	**65.58 |||||||||**	**-0.58**	**0.718**	**0.922**
B:GLY 70	74.38	0.14 |	-0.00	0.82	1.01
B:ILE 31	77.95	41.24 ||||||	0.66	0.968	0.961
B:LYS 32	172.11	69.62 |||||	-0.63	1.122	1.128
B:LYS 34	86.57	0.53 |	-0.02	1.26	1.219
B:ASP 72	70.94	0.32 |	-0.01	0.877	1.121
B:LYS 74	150.84	8.95 |	-0.33	1.139	1.419
B:HIS 75	93.50	36.24 ||||	0.14	1.008	1.358
B:TRP 76	81.71	49.42 |||||||	0.62	0.889	1.104
**B:LYS 88***	**128.28**	**40.17 ||||**	**-0.37**	**0.867**	**0.895**
B:GLN 96	117.33	74.81 |||||||	0.53	1.916	1.864
B:ALA 98	56.29	36.23 |||||||	0.58	1.245	1.228
B:TRP 99	153.54	38.62 |||	0.08	1.084	1.017
**B:ARG 100***	**73.16**	**53.41 |||||||||**	**-1.03**	**0.948**	**0.907**
B:PHE 101	83.50	43.01 ||||||	0.69	0.869	0.844
**B:ARG 114***	**186.97**	**121.28 ||||||||**	**-2.15**	**0.973**	**1.3**
B:THR 117	187.35	11.54 |	0.05	4.443	3.927

*Residues involved in hydrogen bonds or salt bridges

Table S4: Details of p75NTR residues involved in the interface surface of the proNGF/2p75NTR complex (PDB_ID 3IJ2)

P75NTR Chain: residue	Accessible Surface Area ASA(Å^2)	Buried Surface Area BSA(Å^2) ; Buried area percentage, one bar per 10%	Solvation energy effect Δ^iG (kcal/mol)	RMSF p75NTR with proNGF WT	RMSF p75NTR with proNGF R100W
X:LEU 40	115.69	47.53 |||||	0.76	1.85	1.558
X:ASP 41	73.15	11.96 ||	0.05	1.949	1.571
X:SER 42	83.50	70.13 |||||||||	-0.00	1.528	1.478
X:VAL 43	71.57	28.28 ||||	0.45	1.647	1.478
X:MET 67	112.29	64.76 ||||||	1.23	1.076	1.081
**X:SER 68***	**59.12**	**17.73 |||**	**-0.17**	**1.067**	**1.049**
X:PRO 70	84.14	45.63 ||||||	0.73	0.993	0.963
X:CYS 71	9.86	3.71 ||||	-0.03	0.992	0.973
X:TYR 83	178.10	51.09 |||	0.50	1.157	1.396
X:LEU 106	24.93	13.71 ||||||	0.09	1.154	1.385
X:VAL 107	62.56	32.98 ||||||	-0.08	1.239	1.63
X:PHE 108	127.82	54.14 |||||	0.87	1.285	1.634
X:ASP 128	92.52	22.54 |||	-0.26	1.861	1.868
X:GLU 129	89.11	5.28		-0.09	1.605	1.706
X:ASN 131	33.30	1.74		-0.02	1.291	1.324
**X:VAL 133***	**79.03**	**57.99 ||||||||**	**0.57**	**1.047**	**1.151**
**X:ASP 134***	**57.38**	**33.89 ||||||**	**-0.21**	**1.162**	**1.106**
X:PRO 135	64.73	47.14 ||||||||	0.75	1.159	1.261
X:LEU 137	82.44	37.32 |||||	0.60	1.841	1.476
X:ASN 20	66.45	25.67 ||||	-0.01	2.149	2.059
X:LEU 21	91.88	39.88 |||||	0.41	1.931	1.961
X:GLY 22	7.71	2.01 |||	0.03	1.671	1.717
**X:ASP 41***	**73.15**	**33.55 |||||**	**-0.54**	**1.949**	**1.571**
X:VAL 49	85.98	4.43		0.07	2.075	2.723
X:SER 68	59.12	8.13 ||	-0.09	1.067	1.049
X:ALA 69	38.22	30.19 ||||||||	0.48	0.976	0.972
X:PRO 70	84.14	38.51 |||||	0.62	0.993	0.963
X:VAL 72	64.99	55.12 |||||||||	0.88	1.098	1.223
X:GLU 73	46.49	23.85 ||||||	-0.25	1.143	1.475
X:ALA 74	56.24	51.85 ||||||||||	0.79	1.167	1.181
**X:ASP 75 ***	**88.69**	**51.64 ||||||**	**-0.29**	**1.057**	**1.025**
X:ASP 76	28.44	1.60		-0.02	0.949	0.914
X:VAL 78	77.67	18.74 |||	0.30	1.004	0.968
X:LEU 106	24.93	3.07 ||	-0.04	1.154	1.385
X:VAL 107	62.56	1.17		0.02	1.239	1.63
**X:GLU 119***	**75.22**	**12.25 ||**	**-0.15**	**1.328**	**1.781**
X:PRO 122	65.91	8.51 ||	0.14	1.573	1.885
X:GLU 123	173.33	7.62		0.01	1.782	2.004
X:THR 125	24.20	9.54 ||||	0.15	1.549	1.585
X:PRO 135	64.73	17.58 |||	0.28	1.159	1.261
X:CYS 136	23.72	23.55 ||||||||||	-0.07	1.315	1.335
X:LEU 137	82.44	12.27 ||	0.20	1.841	1.476
X:PRO 138	103.34	68.86 |||||||	1.10	2.006	1.753

<table>
<tr><td>X:CYS 139</td><td>25.16</td><td>6.06 |||</td><td>-0.07</td><td>1.98</td><td>1.863</td></tr>
<tr><td>X:VAL 141</td><td>133.07</td><td>15.55 ||</td><td>0.25</td><td>2.823</td><td>2.668</td></tr>
<tr><td>Y:LEU 40</td><td>115.14</td><td>51.02 |||||</td><td>0.82</td><td>1.349</td><td>1.269</td></tr>
<tr><td>Y:ASP 41</td><td>74.01</td><td>23.20 ||||</td><td>0.13</td><td>1.341</td><td>1.108</td></tr>
<tr><td>Y:SER 42*</td><td>83.54</td><td>69.63 |||||||||</td><td>-0.11</td><td>1.255</td><td>1.049</td></tr>
<tr><td>Y:VAL 43</td><td>72.45</td><td>27.46 ||||</td><td>0.44</td><td>1.11</td><td>1.12</td></tr>
<tr><td>Y:MET 67</td><td>111.21</td><td>60.70 ||||||</td><td>1.09</td><td>0.963</td><td>1.052</td></tr>
<tr><td>Y:SER 68*</td><td>57.55</td><td>21.75 ||||</td><td>-0.20</td><td>1.009</td><td>1.063</td></tr>
<tr><td>Y:ALA 69</td><td>36.96</td><td>0.33 |</td><td>0.01</td><td>0.941</td><td>0.939</td></tr>
<tr><td>Y:PRO 70</td><td>84.61</td><td>49.22 ||||||</td><td>0.79</td><td>0.935</td><td>0.895</td></tr>
<tr><td>Y:CYS 71</td><td>10.04</td><td>4.69 |||||</td><td>-0.04</td><td>0.908</td><td>0.851</td></tr>
<tr><td>Y:TYR 83</td><td>181.00</td><td>44.16 |||</td><td>0.41</td><td>1.228</td><td>1.269</td></tr>
<tr><td>Y:LEU 106</td><td>26.23</td><td>12.47 |||||</td><td>0.06</td><td>1.149</td><td>1.102</td></tr>
<tr><td>Y:VAL 107</td><td>64.08</td><td>37.99 ||||||</td><td>0.00</td><td>1.251</td><td>1.299</td></tr>
<tr><td>Y:PHE 108</td><td>127.37</td><td>54.25 |||||</td><td>0.87</td><td>1.32</td><td>1.376</td></tr>
<tr><td>Y:GLN 111</td><td>81.32</td><td>6.26 |</td><td>-0.07</td><td>1.479</td><td>1.441</td></tr>
<tr><td>Y:ASP 128</td><td>95.40</td><td>17.66 ||</td><td>-0.22</td><td>1.789</td><td>1.935</td></tr>
<tr><td>Y:ASN 131</td><td>33.27</td><td>0.58 |</td><td>-0.01</td><td>1.209</td><td>1.302</td></tr>
<tr><td>Y:VAL 133*</td><td>79.16</td><td>52.16 |||||||</td><td>0.52</td><td>1.281</td><td>1.069</td></tr>
<tr><td>Y:ASP 134*</td><td>58.57</td><td>32.38 ||||||</td><td>-0.17</td><td>1.281</td><td>1.111</td></tr>
<tr><td>Y:PRO 135</td><td>64.39</td><td>50.41 ||||||||</td><td>0.81</td><td>1.24</td><td>1.047</td></tr>
<tr><td>Y:LEU 137</td><td>83.73</td><td>44.34 ||||||</td><td>0.71</td><td>1.572</td><td>1.514</td></tr>
<tr><td>Y:GLU 143</td><td>128.67</td><td>7.36 |</td><td>-0.12</td><td>3.925</td><td>3.973</td></tr>
<tr><td>Y:ASN 20</td><td>66.72</td><td>19.54 |||</td><td>0.09</td><td>1.884</td><td>1.932</td></tr>
<tr><td>Y:LEU 21</td><td>91.60</td><td>50.77 ||||||</td><td>0.59</td><td>1.599</td><td>1.526</td></tr>
<tr><td>Y:GLY 22</td><td>8.10</td><td>1.68 |||</td><td>0.03</td><td>1.417</td><td>1.289</td></tr>
<tr><td>Y:ASP 41*</td><td>74.01</td><td>25.48 ||||</td><td>-0.42</td><td>1.341</td><td>1.108</td></tr>
<tr><td>Y:VAL 49</td><td>86.54</td><td>11.46 ||</td><td>0.18</td><td>1.907</td><td>1.925</td></tr>
<tr><td>Y:SER 68</td><td>57.55</td><td>9.51 ||</td><td>-0.10</td><td>1.009</td><td>1.063</td></tr>
<tr><td>Y:ALA 69</td><td>36.96</td><td>30.43 |||||||||</td><td>0.49</td><td>0.941</td><td>0.939</td></tr>
<tr><td>Y:PRO 70</td><td>84.61</td><td>35.38 |||||</td><td>0.56</td><td>0.935</td><td>0.895</td></tr>
<tr><td>Y:VAL 72</td><td>64.60</td><td>57.07 |||||||||</td><td>0.91</td><td>0.908</td><td>0.851</td></tr>
<tr><td>Y:GLU 73</td><td>46.70</td><td>19.27 |||||</td><td>-0.25</td><td>1.112</td><td>0.885</td></tr>
<tr><td>Y:ALA 74</td><td>56.11</td><td>53.65 ||||||||||</td><td>0.74</td><td>1.235</td><td>1</td></tr>
<tr><td>Y:ASP 75*</td><td>88.89</td><td>53.91 |||||||</td><td>-0.36</td><td>1.221</td><td>0.948</td></tr>
<tr><td>Y:ASP 76</td><td>28.46</td><td>1.10 |</td><td>-0.01</td><td>1.069</td><td>0.874</td></tr>
<tr><td>Y:VAL 78</td><td>77.57</td><td>23.58 ||||</td><td>0.38</td><td>0.916</td><td>0.99</td></tr>
<tr><td>Y:LEU 106</td><td>26.23</td><td>3.07 ||</td><td>-0.04</td><td>1.149</td><td>1.102</td></tr>
<tr><td>Y:VAL 107</td><td>64.08</td><td>1.00 |</td><td>0.02</td><td>1.251</td><td>1.299</td></tr>
<tr><td>Y:GLU 119*</td><td>74.32</td><td>12.12 ||</td><td>-0.15</td><td>1.292</td><td>1.429</td></tr>
<tr><td>Y:PRO 122</td><td>65.91</td><td>8.34 ||</td><td>0.13</td><td>1.623</td><td>1.774</td></tr>
<tr><td>Y:GLU 123</td><td>173.97</td><td>12.69 |</td><td>0.14</td><td>1.879</td><td>1.956</td></tr>
<tr><td>Y:THR 125</td><td>24.05</td><td>11.21 |||||</td><td>0.18</td><td>1.591</td><td>1.463</td></tr>
<tr><td>Y:PRO 135</td><td>64.39</td><td>13.97 |||</td><td>0.22</td><td>1.24</td><td>1.047</td></tr>
<tr><td>Y:CYS 136*</td><td>23.69</td><td>23.69 ||||||||||</td><td>-0.06</td><td>1.314</td><td>1.152</td></tr>
<tr><td>Y:LEU 137</td><td>83.73</td><td>10.63 ||</td><td>0.17</td><td>1.572</td><td>1.514</td></tr>
<tr><td>Y:PRO 138</td><td>101.25</td><td>65.49 |||||||</td><td>1.05</td><td>1.826</td><td>1.77</td></tr>
<tr><td>Y:CYS 139</td><td>25.09</td><td>4.40 ||</td><td>-0.05</td><td>1.986</td><td>1.831</td></tr>
<tr><td>Y:VAL 141</td><td>136.11</td><td>13.05 |</td><td>0.21</td><td>2.778</td><td>2.647</td></tr>
</table>

*Residues involved in hydrogen bonds or salt bridges.

Figure S1: Plots of Root-Mean-Square Fluctuation of the backbone of the two chains of unbound NGF: (a) NGF_A WT and R100W, (b) NGF_B WT and R100W.

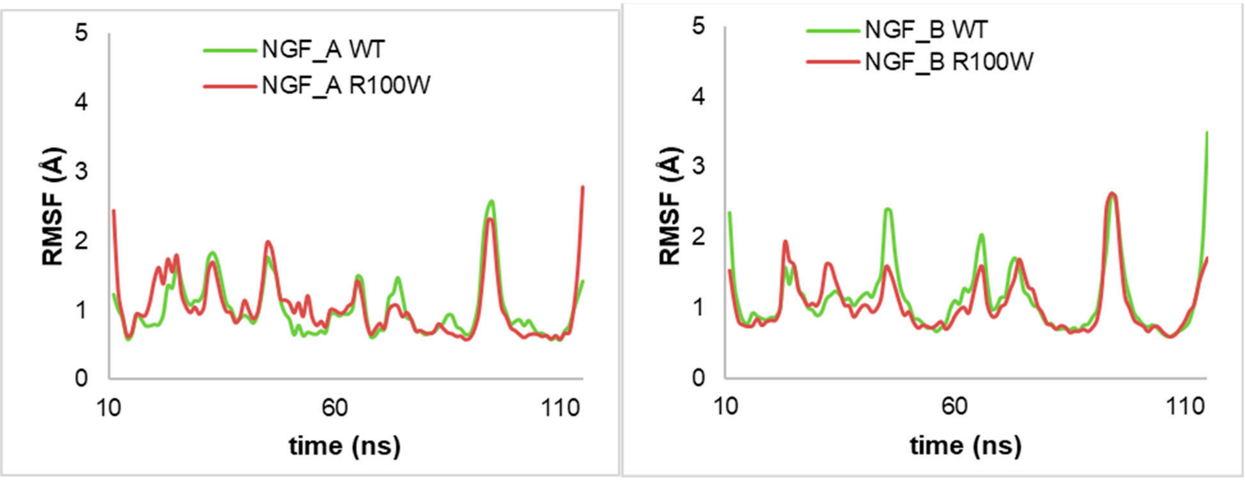

(a) (b)

Figure S2: Plots of Root-Mean-Square Fluctuation of the backbone of the two chains of unbound proNGF: (a) proNGF_A WT and R100W, (b) proNGF_B WT and R100W.

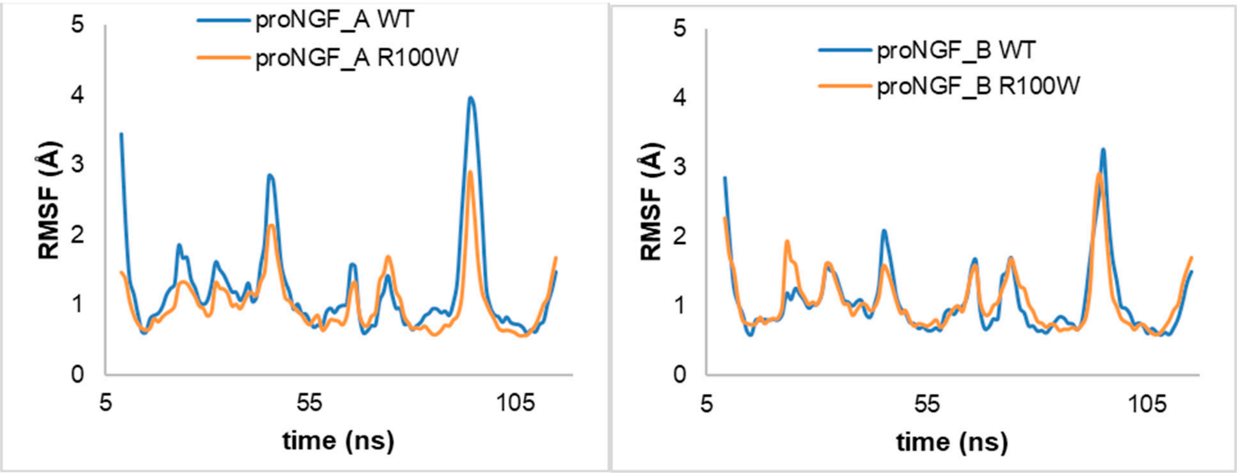

(a) (b)

Figure S3: Root Mean-Square Deviations plots of: (a) NGF and (b) proNGF in complex with p75NTR.

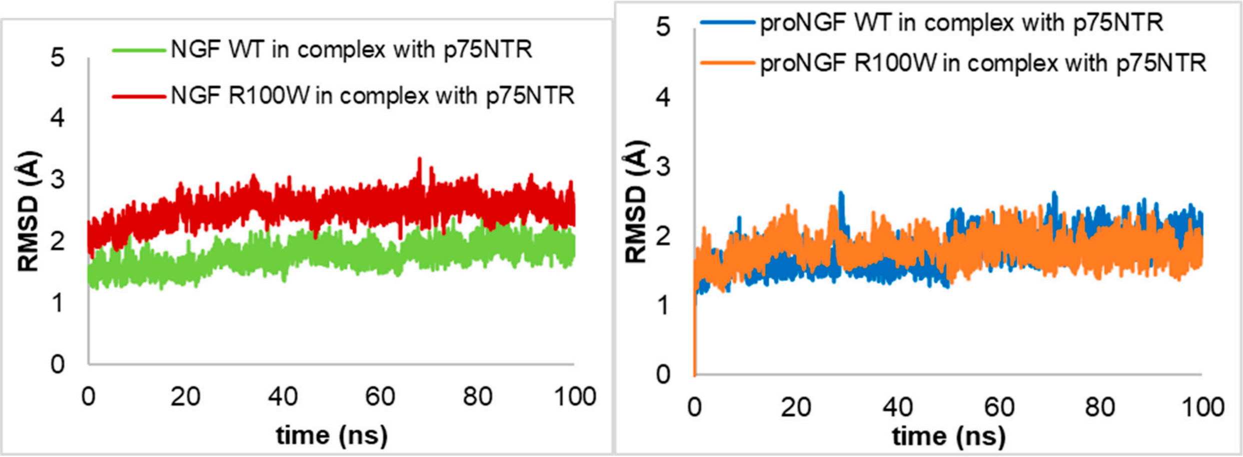

(a) (b)

Figure S4: Root Mean-Square Deviations plots of the complexes: p75NTR in complex with NGF (a), p75NTR_X (b) and p75NTR_Y (c) in complex with proNGF.

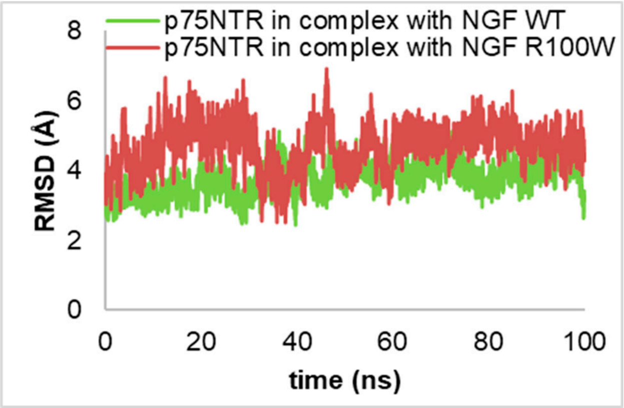

(a)

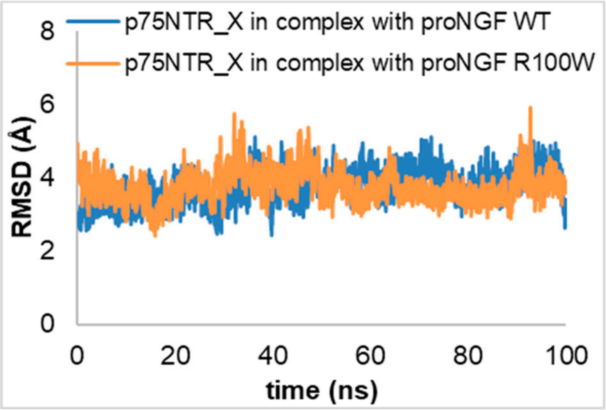

(b)

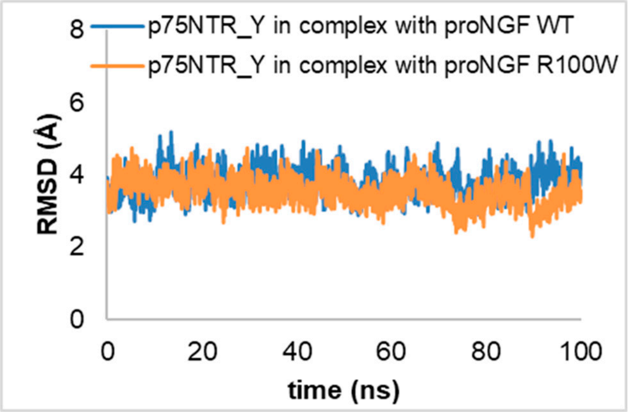

(c)

Figure S5: SPR analysis of hNGF R100W binding to NGF receptors: A) binding of 1.7–15 nM hNGF R100W to TrkA (immobilized at 8860 RU) and B) to p75NTR (immobilized at 4320 RU). All curves were blank subtracted.

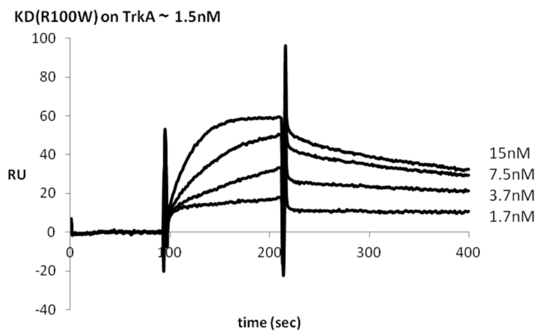

(**a**)

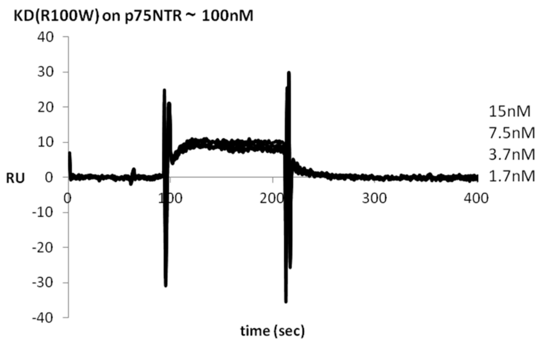

(**b**)
